# Microstructure Evolution and Deformation Mechanisms of As-Cast Antibacterial Ti6Al4V-5Cu Alloy for Isothermal Forging Process

**DOI:** 10.3390/ma15093349

**Published:** 2022-05-06

**Authors:** Solomon Kerealme Yeshanew, Chunguang Bai, Qing Jia, Tong Xi, Zhiqiang Zhang, Diaofeng Li, Zhizhou Xia, Rui Yang, Ke Yang

**Affiliations:** 1 Shi-Changxu Innovation Center for Advanced Materials, Institute of Metal Research (IMR), Chinese Academy of Sciences (CAS), Shenyang 110016, China; solomon19b@imr.ac.cn (S.K.Y.); qjia@imr.ac.cn (Q.J.); txi@imr.ac.cn (T.X.); zqzhang@imr.ac.cn (Z.Z.); dfli16b@imr.ac.cn (D.L.); zzxia15s@imr.ac.cn (Z.X.); ryang@imr.ac.cn (R.Y.); 2 School of Materials Science and Engineering, University of Science and Technology of China, Shenyang 110016, China

**Keywords:** Ti6Al4V-5Cu alloy, hot deformation, constitutive equation, microstructure, processing map

## Abstract

The hot workability behavior of antibacterial Ti6Al4V-5Cu alloy was investigated using a hot compression experiment in the temperature range of 790–1040 °C and strain rate of 10^−3^–10 s^−1^ with a strain of 0.4. The deformation behavior of the alloy was characterized by Gleeble 3800 compression experiment, and the relationship among deformed microstructures and deformation parameters was established. The deformations of Ti6Al4V-5Cu alloy were temperature and strain rate-dependent. Higher temperature and lower strain rate made power dissipation efficiency (η) increase and reach 89%. The activation energies (Q) in the dual-phase (α + β) and single β phase regions were calculated as 175.43 and 159.03 kJ mol^−1^, respectively. In the dual (α + β) phase region, with an increase in strain rate, flow-softening behavior was dominated, however in the single β phase region such as processing at 940 °C. Flow stress increased slightly in which work-hardening behavior was dominated (especially between strain rates of 10^−3^–1 s^−1^). The deformation at various conditions exhibited different stress-strain profiles, providing an insight that work hardening and flow softening coexisted in Ti6Al4V-5Cu alloy. The relative intensity of oscillatory change in flow stress profile decreased as the strain rate decreased. The hot workability of Ti6Al4V-5Cu alloy was also accessed from the viewpoint of the sub-grain structure.

## 1. Introduction

Titanium alloys have been used in many industrial applications such as automotive and aerospace due to their excellent comprehensive properties [1,2]. In order to obtain fine microstructures and favorable properties, a well-designed multistep thermomechanical processing (TMP) is critically needed in the manufacturing of titanium components [3]. Titanium alloys are also widely used in medical areas [4,5,6]. A large number of titanium alloys have been used for human implants, mainly used for artificial joints or as implant and repair material for human hard tissue [7,8,9]. Among titanium alloys, Ti6Al4V alloy is the most applicable due to its excellent mechanical properties and improved corrosion resistance, particularly attractive in aerospace and biomedical applications [4,10]. One of the clinical problems seen in surgical implantation is associated with bacterial infection [7]. Ling Ren et al. [7] showed that Ti6Al4V-xCu alloys with three different copper contents of 1, 3, and 5 wt.% had suitable antibacterial abilities and suitable corrosion resistance. On Ti6Al4V-xCu alloy, the antibacterial behavior was enhanced by an increase in the Cu content [7]. The search for biomaterials that are able to provide for the optimal resistance to infection can be based only on the deep understanding of the interactions between bacteria and biomaterials [11]. Currently, the antibacterial metals have shown great potential as new kinds of biomaterials in which Cu has been used as an antibacterial agent in addition to biodegradable metals such as magnesium and zinc alloys and is also used as an alloying element to adjust the degradation rate [12].

The economic factors that determine the effectiveness of a manufacturing process govern the proper use of materials since the cost-benefit advantage of a particular firm over the global competitive environment is limited by the capability of affording items with minimum cost and longer time use under service conditions. Based on this economic feasibility and justification, processing alloys at high temperatures is more economical and desirable, which provides material reduction to an oxidizing condition [13]. The inherent characteristics of titanium alloys, such as poor thermal conductivity and high reactivity to oxygen, make conventional processing very challenging [14]. Currently, the advanced manufacturing technologies have given the competitive advantages of producing complex and intricate 3D-part geometry of near-net-shaped products that do not require further machining and finishing operation under controlled conditions in a single step, and small lot sizes have been innovated such as additive manufacturing technology [14].

Hot deformation in various practical industrial processes has used hot forging or rolling mechanisms for the manufacturing of semi-finished and finished products of titanium alloys. Mostly, products fabricated by hot processing equipment possess complex setups, tedious processes, and high production costs. The alloy can be processed from a single or dual-phase region, or both depending on the purpose of imparting a set of desired properties on a particular alloy. For instance, the α/β hot working operation gives rise to a structure composed of primary α particles in a matrix of β phase, which contains residual dislocation substructure [15]. Concerning the hot deformation characteristics of titanium alloys, many investigations have been carried out. Most of the hot deformation behavior involves investigating whether there are apparent differences among the alloy flow stress curves acquired in either dual-phase (α + β) and single β-phase regions through analyzing the dependence of flow stress to temperature, strain rate, and microstructural evolutions, as Momeni and Abbasi [10] investigated the influence of hot working conditions on the flow behavior of Ti6Al4V alloy in single-phase and dual-phase regions. It is known that the flow stress level increases with strain rate and decreases on temperature [10,16]. Peng et al. [17] studied the effect of processing parameters on the flow stress behavior and microstructure evolution during the hot compression of as-cast Ti60 alloy. According to their investigation, the alloy flow behavior induced higher flow softening in the dual-phase region than that in the single-phase region. They elaborated that the occurrences of such flow-softening condition were raised due to the breaking up of lamellar α, deformation heating, flow localization, and free-surface cracking. Generally, a further increase in the strain makes the flow stress decrease progressively, which is particularly evident at higher temperatures and under high strain rate conditions, indicating that flow softening arises due to dynamic recrystallization [18,19,20,21]. According to Guo et al. [22], the dynamic transformation process was described as the primary source of flow softening in titanium alloys, whereas the dynamic coarsening was mentioned as the primary source of flow hardening at low strain rates. The coarsening in titanium alloys is controlled by bulk diffusion propensity and can occur more freely during the longer deformation time. Such coarsening behavior gives rise to the loss of grain boundaries, which act as the sources of deformation at elevated temperatures. The texture variation of titanium alloys can also provide flow softening or hardening, especially in the α phase [22]. The workability of titanium alloys is also related to the sub-grain structure, stress-induced phase transformation, and texture [23]. Controlling the morphology of the α phase is important to improve the properties of Ti6Al4V alloy and other (α + β) titanium alloys [24]. The volume fraction of the β phase is very critical for super plasticity since a smaller fraction (20%) is essential for maintaining a stable fine-grained structure while a higher fraction increases the undesirable α/β boundaries [25].

There have been many studies on the hot deformation behavior of titanium alloys in order to optimize the processing conditions through constructing hot deformation processing maps. The hot deformation of Ti6Al4V alloy indicated that the flow stress was strongly dependent on both temperature and strain rate [26]. During the hot deformation process, plastic unstable behaviors such as adiabatic shear band, flow localization, and cracking exist, which can affect material properties and can be identified by processing maps through combining microstructure analysis with alloy instability and stability regions [25,27,28]. The optimized process did not produce deformation defects involving shear bands, cavities, and other non-uniformities in metal forming [28]. S. Bruschi [29] investigated the hot workability of Ti6Al4V alloy by means of a hot compression experiment in the temperature range of 880–950 °C and 1–50 s^−1^ strain rate, and described that the recommended maximum strain rate was 10 s^−1^. Zheng Ma et al. [27] investigated the thermomechanical compression of hot forged Ti6Al4V-5Cu alloy through optimizing its microstructure and hot working process. Globularization of α phase during hot deformation of Ti6Al4V alloy was the major microstructure change that was considered to be responsible for the flow softening regardless of the morphology of constituent phases in the dual-phase region [10]. During the hot deformation process at a high strain rate (1–10 s^−1^) and low temperature around the lowest range of hot deformation, titanium alloys had some micro-cracks, inhomogeneous deformation, macro-zone, and non-uniform microstructure [30]. Therefore, this work involved in investigating the hot deformation behavior of as-cast antibacterial Ti6Al4V-5Cu alloy, aimed at reducing the energy barriers to facilitate the easiness of alloy hot workability through characterizing and investigating the microstructure evolution and deformation mechanisms by means of Gleeble 3800 thermomechanical simulation system in the temperature range of 790–1040 °C with 50 °C intervals at the strain rate range of 10^−3^–10 s^−1^ with a total strain of 0.4, and finally generated a processing window through establishing relationship among deformation parameters.

## 2. Materials and Methods

**Figure 1:**
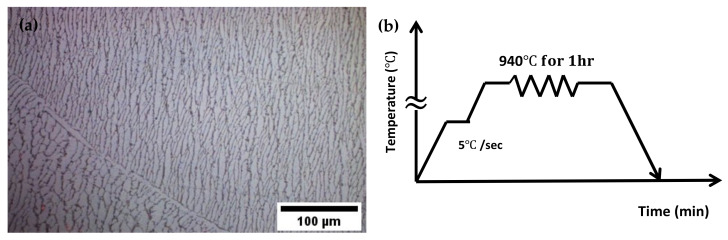
(**a**) Microstructure of Ti6Al4V-5Cu alloy after beta annealing treatment, (**b**) schematic presentation of beta annealing for hot deformation tests.

The Ti6Al4V–5Cu alloy used in this investigation was produced from commercial-grade titanium, aluminum, vanadium, and high-purity Cu (99.99%). The alloy was produced by arc melting using a non-consumable tungsten electrode and water cooling copper crucible in an argon atmosphere. To ensure their chemical homogeneity, the ingots were re-melted many times. After preparing an appropriate alloy element mix-ratio, an ingot of 1200 mm in height with 360 mm diameter was cast in an arc melting casting machine in an argon atmosphere. The casting machine was built using a copper hearth crucible allowing water to circulate for solidification of the casted structures. Finally, a 15 mm-thick pre-form sample was sliced from the initial casted ingots for various engineering applications. Hence, an initial Ø360 mm as-cast Ti6Al4V-5Cu alloy ingot with 15 mm thick was used, which is fabricated as a sliced strip in the Institute of Metal Research, Chinese Academy of Sciences (IMR-CAS). Before the compression test, rectangular alloy samples sectioned from the original round disk were subjected to annealing heat treatment at 940 °C for 1 h. in a furnace with 5 °C/minute heating capacity (Figure 1b). The heat-treated alloy samples were immediately exposed to furnace cooling at a slower rate in the furnace. The initial microstructure of Ti6Al4V-5Cu alloy observed by optical microscope after beta annealing treatment is shown in Figure 1a. To characterize the hot deformation behavior of Ti6Al4V-5Cu alloy processed at various strain rates (i.e., 10^−3^–10 s^−1^) with a total strain of 0.4, cylindrical compression samples of Ø10 mm in diameter by 12 mm in height were sectioned on the beta annealed titanium alloy from its longitudinal direction. A thin thermocouple at the middle length of the samples was attached within a 2 mm distance to measure the distribution of temperature while the machine was under operation during compression. During the deformation process, the sample was held for 5 min prior to each successive deformation step. In order to reduce the friction between the sample and the anvil, graphite was used.

The specimens for crystallographic observation were sectioned from the compressed sample parallel to the compression direction. The specimens were ground sequentially using 150, 800, and 2000 grits of SiC papers. Following grinding, polishing has performed on a fabric polishing cloth using SiO_2_ colloidal suspended water at a polishing rate of 300 rpm and rinsed by water. After the samples were dried using hot compressor air and cleaned by ethanol, ultrasonic cleaning, and finally etching were performed to ensure no remnants of colloidal suspension on the sample surfaces. The samples were etched in a reagent proportion of 13 mL HF, 26 mL HNO_3,_ and 100 mL H_2_O. Optical microscope (OM) and scanning electron microscope (SEM) were used to observe the microstructures of samples.

## 3. Results

### 3.1. True Stress—Strain Behavior

**Figure 2:**
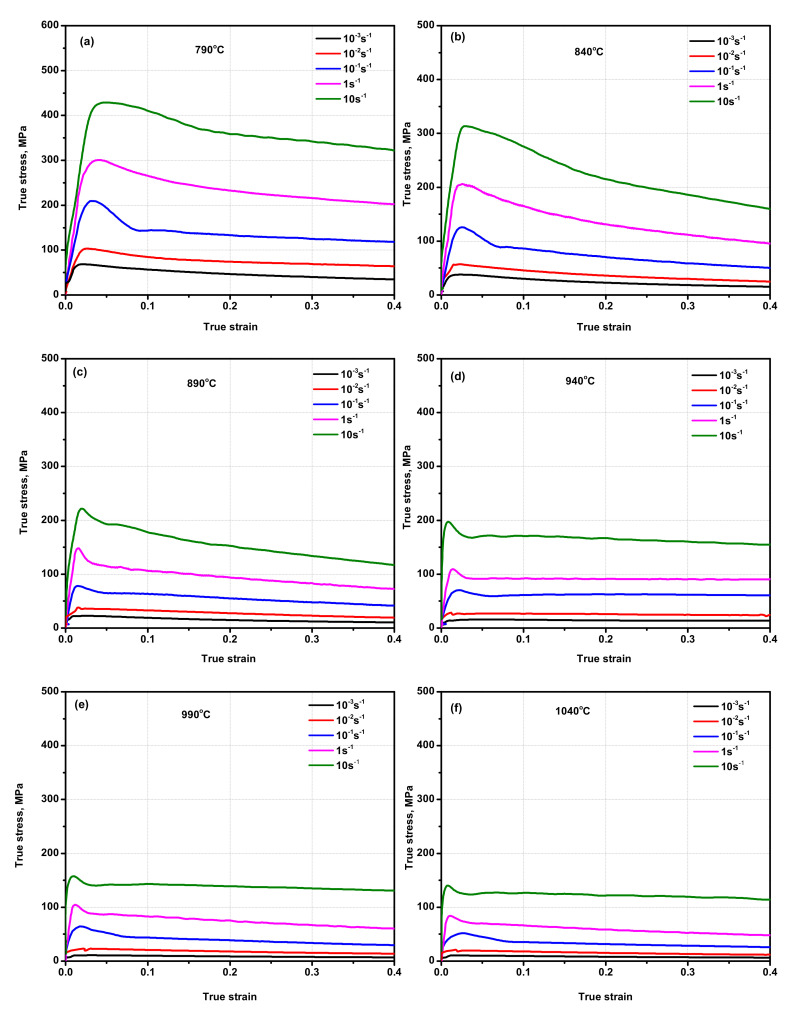
True stress-strain relationships of Ti6Al4V-5Cu alloy during hot compression experiment performed at various strain rates and temperatures, (**a**) 790 °C, (**b**) 840 °C, (**c**) 890 °C, (**d**) 940 °C, (**e**) 990 °C, and (**f**) 1040 °C.

**Figure 3:**
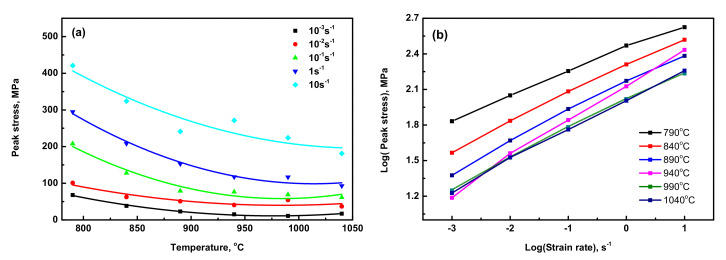
(**a**) Effect of change in temperature on peak flow stress of Ti6Al4V-5Cu alloy at various strain rates 10^−3^–1 s^−1^; (**b**) effect of strain rate on peak flow stress in the temperature range of 790–1040 °C.

The flow stress curves obtained in the dual-phase (α + β region) and single-phase (β region) of Ti6Al4V-5Cu alloy are presented in Figure 2. Figure 2a shows the true stress-strain relationship of Ti6Al4V-5Cu alloy obtained during hot compression carried out at a temperature of 790 °C in various strain rates. Ti6Al4V-5Cu alloy showed various stress-strain profiles during deformation conducted under various processing parameters. For instance, during deformation at a strain rate of 10^−1^ s^−1^, the alloy had a peak stress value of 207.74 MPa at a true strain of 0.025. After flow stress reached its maxima, it dropped down until it reached 148.00 MPa and then stepped down in a lower degree of inclination until the desired level of deformation was achieved. On deformation at a strain rate of 10^−2^ s^−1^, the flow stress initially increased until it reached the maximum of 100.93 MPa, and then declined immediately until the alloy was completely deformed. From the true strain-stress relationship curve, we can conclude that the flow stress increased as the strain rate was increased. At 840 °C and in strain rate of 10^−1^ s^−1^ (Figure 2b), the alloy peak stress reached 127.98 MPa at a true strain of 0.025. Under this deformation condition, flow softening also existed. Likewise, on the last experiment at 1040 °C in strain rate of 10^−3^, 10^−2^, 10^−1^, 1, and 10 s^−1^, true stress-strain relationship curves were plotted as shown in Figure 2f. On a sample deformed at a strain rate of 0.001 s^−1^, initially, the flow stress increased until it reached its maximum peak stress and then declined slightly. Such decline in flow stress in a certain strain rate range occurred due to the existence of dynamic recrystallization and internal micro-cracks. Form deformation at various strain rates, the alloy possessed a thermal expansion. Through referring to true strain-stress curves, the flow stress profile was challenging to identify in the initial strain region due to the thermal expansion behavior of the alloy; however, the alloy attained the maximum peak stress of 16.85 MPa at a true strain of 0.04. For the case of deformation at a strain rate of 10^−2^ s^−1^, the alloy began by nearly vertical flow stress increment until it reached the maximum peak stress of 36.79 MPa. Another feature of compression carried out at various deformation variables with 0.001 s^−1^ and 0.01 s^−1^ strain rates indicated that the alloy possessed dynamic recrystallization and flow-softening behavior. It is also possible to notice that as the deformation strain rate increased, the existence of flow-softening behavior became dominated. During deformation at slower strain rates (i.e., 0.001 and 0.01 s^−1^), the presence of a wavy strain-stress profile provided insight into the co-existence of work hardening and flow softening in Ti6Al4V-5Cu alloy. The flow softening during hot working of dual-phase alloys with lamellar microstructure could occur by rotation of lamellar colonies toward the softer orientations and/or by slip transmission across α/β interphase boundaries [31,32]. The same flow characteristics occurred in Ti6Al4V-5Cu alloy since flow stress initially increased rapidly due to work hardening, increasing with the rise of strain rate and decline of temperature. Under higher deformation reduction, annihilation, and intermixed nature of tangled dislocations within substructures in the alloy were accelerated, which brought a way for the formation of high dislocation density. With increase in the strain, sub-grain structures were transformed by accumulated dislocation tangles, which promoted the transformation of sub-grain structures into recrystallized grains. The flow stress decreased with the rise of the deformation temperature in the range of 790–890 °C (Figure 3a), whereas deformation above 890 °C and in the single β phase region made the peak flow stress fluctuate with change in temperature and strain rate though mostly such fluctuation was assumed negligible.

The maximum true strain at which the peak stress reached was calculated as 0.039 (Table 1). The other feature of deformation at a strain rate of 0.01 s^−1^, as shown in Figure 2, can be easily noticed that true stress-strain curves revealed the occurrence of sharp peak stress in Ti6Al4V-5Cu alloy. Plastic deformation occurred if the stress exceeded the yield point. Figure 3a shows the effect of change in temperature on peak flow stress at various strain rates, which indicates that the peak flow stress was varied depending on the deformation temperature. Figure 3b shows the effect of strain rate on the peak flow stress in the temperature range of 790–1040 °C. Generally, for Ti6Al4V alloy, the peak flow stress increased as the deformation strain rate was increased. Due to the addition of copper, the effect of change in the peak stress with strain rate was found different in the dual (α + β) phase region and single β phase region. In the temperature range of 790 to 890 °C, the peak flow stress decreased with an increase in the temperature, whereas at temperatures above 890 °C, the flow stress did not have a constant relationship with the change in temperature. Thus, an indication of the peak flow stress of Ti6Al4V-5Cu alloy was temperature and strain rate sensitive. Such differences in flow stresses occurred due to the variation of copper content under various processing conditions, especially in the single β phase region. According to the scanning electron microscope (SEM) mapping, the composition and distribution of copper in Ti6Al4V-5Cu alloy has contributed a significant role to improve the flow stress tendency so as to provide an enhanced overall plasticity behavior. The peak flow stress variations under different processing conditions were obviously related to the occurrences of slightly different copper content for a shorter duration when the alloy compression reached the plastic zone. In the temperature range of 890–1040 °C, the acquired peak flow stress of Ti6Al4V-5Cu alloy had a direct relationship with the change in copper content, especially the β phase morphology.

### 3.2. Constitutive Analysis

A constitutive equation describes the path chosen by a given system to reach the applied strain rate [33]. Mathematically, the effects of temperature and strain rate are described in terms of a set of relationships called the Zenner–Hollomon parameter, where Z is given by the equation as follows [4,10,27,34]:

(1) $$Z=\bar{\varepsilon }\exp(Q/\mathrm{RT})$$

(2) $$\bar{\varepsilon }={\;A}_{1}\sigma^{n_{1}}\exp(-Q/\mathrm{RT})$$

(3) $$\bar{\varepsilon }={\;A}_{2}\exp(\beta \sigma )\exp(-Q/\mathrm{RT})$$

(4) $$\bar{\varepsilon }=A{\left[\sin h\left(\alpha \sigma \right)\right]}^{n}\exp(-Q/\mathrm{RT})$$

where σ is the flow stress, $\bar{\varepsilon }$ is the strain rate, Q is the apparent activation energy (kJ/mol), R is the universal gas constant (8.314 Jmol^−1^ K^−1^), T is the absolute temperature (in Kelvin), (A_1_, A_2_, n_1_, n, β, and (α = β/$n_{1}$)) are material constants. A expresses the material constant for the particular strain, n is the stress exponent, which is defined as the reverse of strain rate sensitivity exponent, m [10].

Equation (2) is used at low stress level (i.e., ασ <0.8), and Equation (3) is used at high stress level (i.e., ασ ≥1.2). The hyperbolic sine law in Equation (4) can be used for both low stress and high stress levels by summing the material flow during deformation as a thermally activated process [34,35]. The material constants, $A_{1}$, $A_{2}$, A, $n_{1}$, n, β and α, should be identified first for analyzing the flow behavior of the alloy. Taking natural logarithm of Equations (2) and (3) gives [27]:

(5) $$\ln\bar{\varepsilon }=\ln A_{1}+n_{1}\ln\sigma -(Q/\mathrm{RT})$$

(6) $$\ln\bar{\varepsilon }=\ln A_{2}+\beta \sigma -(Q/\mathrm{RT})$$

In order to determine the relationship between either the value of peak strain, peak stress, and temperature compensated strain rate, $\bar{\varepsilon }$ or Zenner–Hollomon parameter Z, the first step is to determine the apparent activation energy. The apparent activation energy describes the activation barrier that atoms need to conquer to follow the deformation procedure [10]. Taking the natural logarithm of Equation (4) yields [27]:

(7) $$\ln\bar{\varepsilon }=\mathrm{lnA}+\mathrm{nln}(\sinh(\alpha \sigma ))-Q/\mathrm{RT}$$

Equation (7) can be described as follows [27]:

(8) $$\ln(\sinh(\alpha \sigma ))=\ln\bar{\varepsilon }/n-\mathrm{lnA}/n+Q/\mathrm{RT}$$

(9) $$Q=\mathrm{nR}\partial \ln(\sinh(\alpha \sigma ))/\partial T^{-1}$$

**Figure 4:**
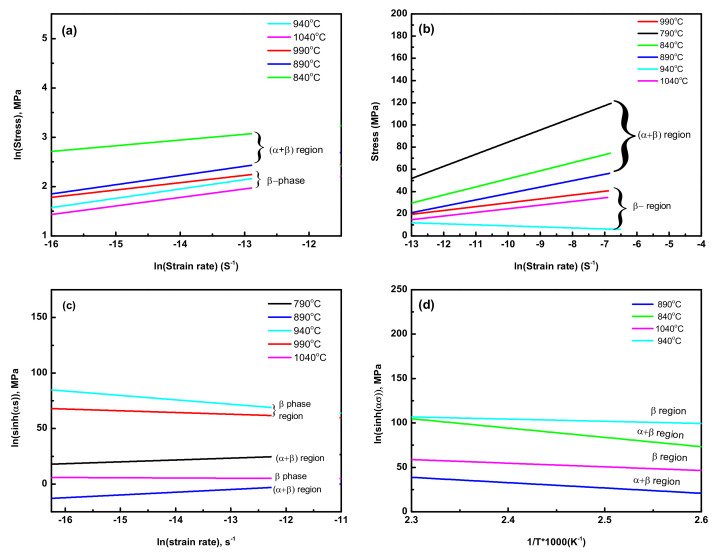
Typical functional relationship plot between ln($\bar{\varepsilon }$) and ln(σ) to determine the material constant, n_1_, at a strain rate of 0.01 s^−1^ (**a**), the functional relationship between ln($\bar{\varepsilon }$) and σ to determine the material constant, β, at a strain rate of 0.1 s^−^^1^ (**b**), the relationship plot between ln(sinh(ασ)) and ln($\bar{\varepsilon }$) to calculate n at a strain rate of 0.001 s^−^^1^ (**c**), and variation of flow stress with the inverse of change in temperature to calculate the activation energy, Q, of as-cast Ti6Al4V-5Cu alloy severely deformed by hot compression at 0.01 s^−1^ (**d**).

Through the functional relationship plot between ln($\bar{\varepsilon }$) and ln(σ) (Figure 4a), the average values of ($n_{1}$) were determined for (σ + β) and β phase regions as 0.13 and 0.2, respectively. Using the formula of (α = β/n_1_), the average values of material constant (α) were also calculated as 10.33 and 37.52 for (σ + β) and β phase regions, respectively. From the linear relationship of ln(sinh(ασ)) and ln($\bar{\varepsilon }$) (Figure 4c), the average maximum values of material constant (n) were calculated as 13.79 and 12.3 for (α + β) and β phase regions, respectively. Using the functional relationship plot between ln($\bar{\varepsilon }$) and ln(σ) (Figure 4a), the average values of ($n_{1}$) were determined for σ + β and β-phase region as 0.13 and 0.2, respectively. Using the formula of (α = β/n_1_), the average value of material constant, (α) was also calculated as 10.33 and 37.52 for σ + β and β-phase region, respectively. From the linear relationship of ln(sinh(ασ)) and ln($\bar{\varepsilon }$) (Figure 4c), the average maximum value of the material constant (n) was calculated as 13.79 and 12.3 for the α + β and β phase region, respectively.

### 3.3. Activation Energy (Q)

The activation energy, Q, at different strain rates was calculated. The apparent activation energies required in the (α + β) region and β region were determined as 175.43 kJ/mol and 159.03 kJ/mol, respectively. Therefore, Zenner–Hollomon parameter, Z, can be expressed using the following equation:

For (α + β) phase region:

(10) $$Z=\bar{\varepsilon }\;\exp(175.43/\mathrm{RT})$$

For β-phase region:

(11) $$Z=\bar{\varepsilon }\;\exp(159.03/\mathrm{RT})$$

**Figure 5:**
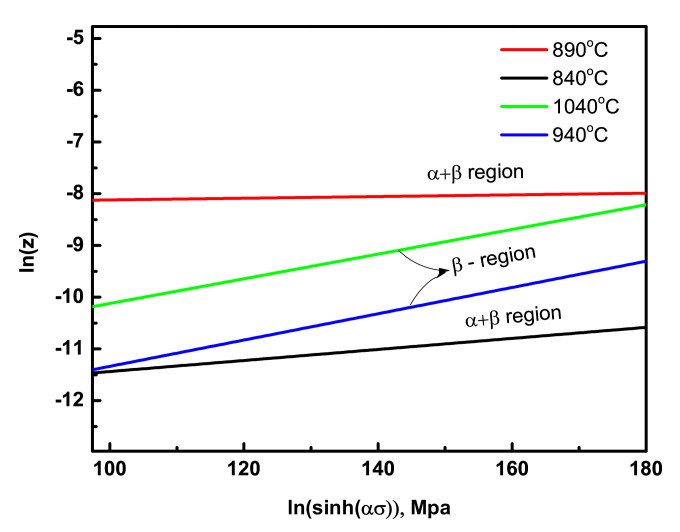
Plot of the relationship between ln(sinh(ασ)) and lnZ at a strain rate of 0.01 s^−1^ for determining the material constant, A.

To find the material constant, A, a relationship analysis between ln(sinh(ασ)) and lnZ at different strain rates was used (Figure 5). In the single β phase region, the activation energy (Q) was usually between 150 and 200 kJ/mol [36]. If the activation energy for hot deformation is close to that for the self-diffusion, the process is a recovery, whereas higher activation energy indicates that the deformation process is governed by dynamic recrystallization [37]. The variation in activation energy for hot deformation arises from different microstructures of titanium alloys processed under different deformation conditions. The maximum apparent activation energy required for each region was previously determined, and the strain rate, $\bar{\varepsilon }$, can be expressed mathematically as a function of flow stress (σ), and temperature (T) in the (α + β) phase region (Equation (12)) and β phase region (Equation (13)) are as follows:

(12) $$\bar{\varepsilon }=0.19[\mathrm{Sinh}(10.33*\sigma ){]}^{13.79}\;\exp(-175.43/\mathrm{RT})$$

(13) $$\bar{\varepsilon }=2.95[\mathrm{Sinh}(37.52*\sigma ){]}^{12.3}\;\exp(-159.03/\mathrm{RT})$$

There have been many studies to investigate the apparent activation energy (Q) for Ti6Al4V alloy, especially in the dual-phase (α + β) and single β-phase regions. The common observation of the noticeable difference between the magnitude of Q for deformation of dual-phase (α + β) alloy and that for single β phase alloy was the presence of variation in phase volume fractions under various deformation conditions. The maximum activation energy, Q, was determined to be 175.43 kJ/mol at a temperature of 790 °C. Though strain hardening constant value at this temperature was found as the lowest in quantity, the energy required for atoms to conquer the internal void or vacant position of neighboring atoms was higher due to the advancement of overall grains in the microstructure to accommodate severe deformation conditions caused by external load. The SEM microstructure study revealed that the globularization process occurred at this temperature through fragmentation of the lamellar grains due to breakage of BCC structural phases found in between primary α of HCP structure. The apparent activation energy, Q, of the single β phase was also calculated to be 159.03 kJ/mol. This clearly indicates that the energy barrier for facilitating deformation at temperatures above β transus was the minimum.

Initially, at the lower deformation temperature, the apparent activation energy (Q) for Ti6Al4V-5Cu alloy decreased with the increase in the deformation temperature until reaching 840 °C. The lowest activation energy was obtained at 840 and 940 °C, which was mostly related to the existence of globularization phenomenon and superplasticity behavior, respectively. A higher energy barrier was observed during deformation at 790 °C. Thus, the higher energy barrier that existed at lower deformation temperatures for Ti6Al4V-5Cu alloy was obviously associated with more energy used to break up and fragment the primary α phase lamellar as deformation continued. After 940 °C, the deformation had a characteristic of step-wise increment in apparent activation energy, which was associated with the dynamic recrystallization mechanism. In the single β-phase region, Ti6Al4V-5Cu alloy acquired the lowest energy barrier at 940 °C.

### 3.4. Strain Rate Sensitivity (m)

The strain rate sensitivity of flow stress is an important parameter in metal-forming processes [20], which reveals a noticeable dependence on temperature, strain rate, and microstructure [24]. The magnitude of strain rate sensitivity is usually in the range of 0.2 to 0.33, with specific values for the β phase titanium alloys being comparable to or slightly higher than those for the α-phase alloys [36]. The responses of the strain rate sensitivity index (m) to strain for various titanium alloys are different [20], which is a typical deformation controlled through dislocation glide characteristics, dynamic spheroidization, and occurrence of grain boundary sliding to some degree [24]. The parameter n is determined as the inverse of strain rate sensitivity m, defined as [29,38]:

(14) $$m=f(\bar{\varepsilon },\;T)={\left[\frac{\partial \;\ln\left(\sigma \right)}{\partial \;\ln\left(\bar{\varepsilon }\right)}\right]}_{T,\bar{\varepsilon }}$$

**Figure 6:**
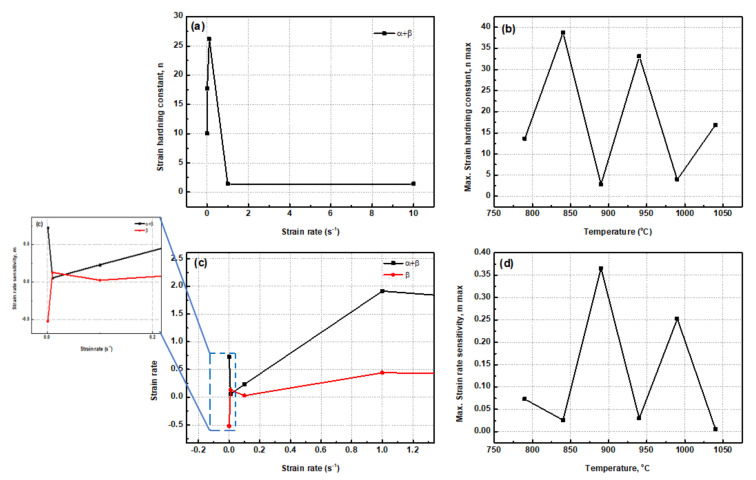
(**a**) Strain hardening constant (n) with change in strain rate (s^−1^), (**b**) maximum strain hardening constant with temperature, (**c**) strain rate sensitivity (m) with strain rate (s^−1^), and (**d**) maximum strain rate sensitivity constant with change in deformation temperature during deformation of Ti6Al4V-5Cu alloy in the strain rate range of 10^−3^ to 10 s^−1^.

The average values of the m of the alloy were calculated as 0.098, 0.16, and 0.26 at strain rates of 0.001, 0.01, and 0.1 s^−1^, respectively. Figure 6b graphically represents the functional relationship between maximum strain hardening constant (n) and change in deformation temperature at various strain rates. The maximum strain hardening constant and strain rate sensitivity of the alloy had a zigzag profile, as seen in Figure 6b,d, respectively. As deformation temperature increased, the relative intensities of n and m were tremendously decreased. The other feature of Ti6Al4V-5Cu alloy deformed in the strain rate range of 10^−3^ to 10^−1^ s^−1^ in dual-phase (α + β) region and single β-phase region revealed that effect of strain rates on m was opposite. For deformation carried out in the dual-phase region, the m initially decreased until strain rate reached 10^−2^ s^−1^ and then increased continuously up to 1 s^−1^. Finally, it was slightly dropped at a higher strain rate such as 10 s^−1^, whereas in the case of single β phase region, the m showed an opposite pattern, i.e., with change in strain rate, it initially increased until strain rate reached 10^−2^ s^−1^ and then decreased up to 10^−1^ s^−1^. In both phase regions, the change of m with respect to strain rate showed a zigzag pattern with the increase in strain rates. From such a relationship, a conclusion can be drawn that Ti6Al4V-5Cu alloy had different strain rate sensitivity (m) effects with regard to change in strain rate, especially at lower rates in the order of magnitude between 10^−3^ and 10^−1^ s^−1^.

**Figure 7:**
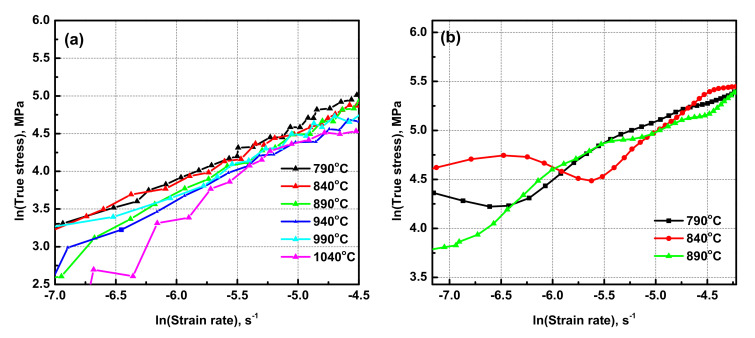
Fluctuation of strain rate sensitivity, m, at strain rates of 1 s^−1^ (**a**) and 10 s^−1^ (**b**).

Based on the relationship between the maximum strain hardening constant and deformation temperature, the lowest strain hardening constant (n) was obtained at 890 °C, 990 and 790 °C, respectively. A higher strain hardening constant (n) was found at 84 °C and 940 °C. The relative fluctuation of strain rate sensitivity index, m, at higher strain rates of 1 and 10 s^−1^ are seen in Figure 7a,b. The relative intensities of strain hardening constant (n) and strain rate sensitivity constant (m) were both tremendously decreased with the increase in deformation temperature. At 840 and 940 °C, Ti6Al4V-5Cu exhibited the least strain rate sensitivity behavior.

### 3.5. Processing Map (PM)

To construct processing map, the dynamic material model (DMM) is mainly used as a method for comparison of different instability criteria to control microstructure during processing [33]. By assuming the thermal working condition as a closed energy manipulator, the whole system can be controlled by two types of energies, which are named input energy (P) and subsequent energy dissipation (content G and co-content J) [30]. The power dissipation maps represent the patterns in which power is dissipated by materials through microstructural changes. The rate of this change is given by a dimensionless parameter called power dissipation efficiency [39]. The value of dissipation efficiency (η) varies with temperature at a given strain rate, which can be calculated by the equation as follows [33,35]:

(15) $$\eta =\frac{2m}{\left(m+1\right)},$$

**Figure 8:**
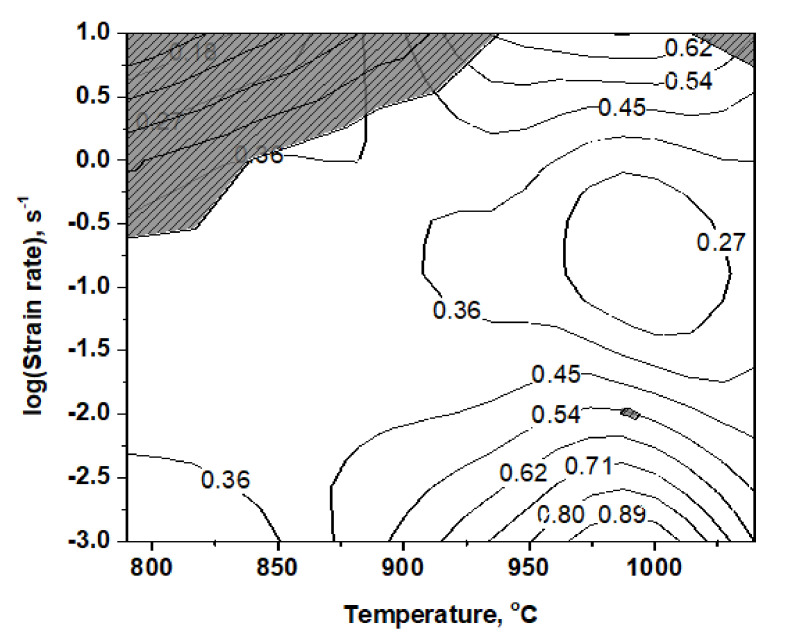
Power dissipation map for Ti6Al4V-5Cu alloy obtained by hot compression experiment in strain rate range of 10^−3^–10 s^−1^ with a strain of 0.4, showing stable and instable regions.

Plotting *η* as a function of strain rate and deformation temperature gives a power dissipation map, which indicates the process of microstructural evolution during hot deformation. A continuum criterion for flow instability can be obtained from the principle of maximum rate of entropy production expressed by [4,40]:

(16) $$\xi (\bar{\varepsilon })=\frac{\partial \ln\left(\frac{2m}{m+1}\right)}{\partial \ln\left(\bar{\varepsilon }\right)}+m<0,$$

where ξ($\bar{\varepsilon }$) is the instability parameter as a function of strain rate and deformation temperature. The contours of different ξ with various strain rates and temperatures constitute an instability map. The processing map (PM) of Ti6Al4V-5Cu alloy was obtained in the temperature range of 790–1040 °C and strain rate range of 10^−3^–10 s^−1^ under a strain of 0.4, as seen in Figure 8.

The contour number represents the power dissipation efficiency (η). In the single β-phase region, η decreased with the increase in strain rate in the range of 10^−3^ s^−1^ to 10^−1^ s^−1^. Whereas at higher strain rates (>1 s^−1^), the η increased with the increase in strain rate. The deformation behaviors of Ti6Al4V-5Cu alloy were strain rate and temperature sensitive. The processing map (PM) of Ti6Al4V-5Cu alloy was obtained after superimposing the instable map on a stable map. The power dissipation efficiency of Ti6Al4V-5Cu alloy reached 89%. Wedge cracking defect was observed using a scanning electron microscope (SEM). The presence of such wedge cracking defect (opening and widening of crack) was decided by the relaxation processes happening at triple junctions where β-phases were present and responsible for restraining grain growth in Ti6Al4V-5Cu alloy. This clearly provided an indication that better deformable characteristics of β phase (BCC) at the triple junctions among subordinating grains should be the main controlling factor for achieving superplasticity. At higher temperatures, the β phase may further exhibit processes that could contribute to the relaxation of stress concentrations involving the dynamic recrystallization process (DRX). Superplasticity involves the sliding of grain boundaries with simultaneous relaxation of stresses generated at the triple junctions of grain boundary by processes involving diffusional flow or plastic deformation [25]. At 840 °C, the volume fraction of β phase was 19.7% (nearly 20%), and this low fraction of β phase permitted many α/α interfaces to slide during hot deformation of Ti6Al4V-5Cu alloy. On PM at different strain rates, it can be noticed that the hot formability region of the alloy is wide. The deformation of Ti6Al4V-5Cu had a limited instability region that existed in the deformation temperature range of 800 to 930 °C and higher strain rates (>1 s^−1^). There had also instability regions obtained at 990 °C during a strain rate of 10^−2^ s^−1^.

### 3.6. Microstructure Evolution and Deformation Mechanisms in Ti6Al4V-5Cu Alloy

In order to improve the processing ability for a new design of titanium alloys, it is vital to understand the deformation mechanisms and microstructural evolution that could develop during deformation. The microstructure and phases of deformed specimens were analyzed using a scanning electron microscope (SEM).

**Figure 9:**
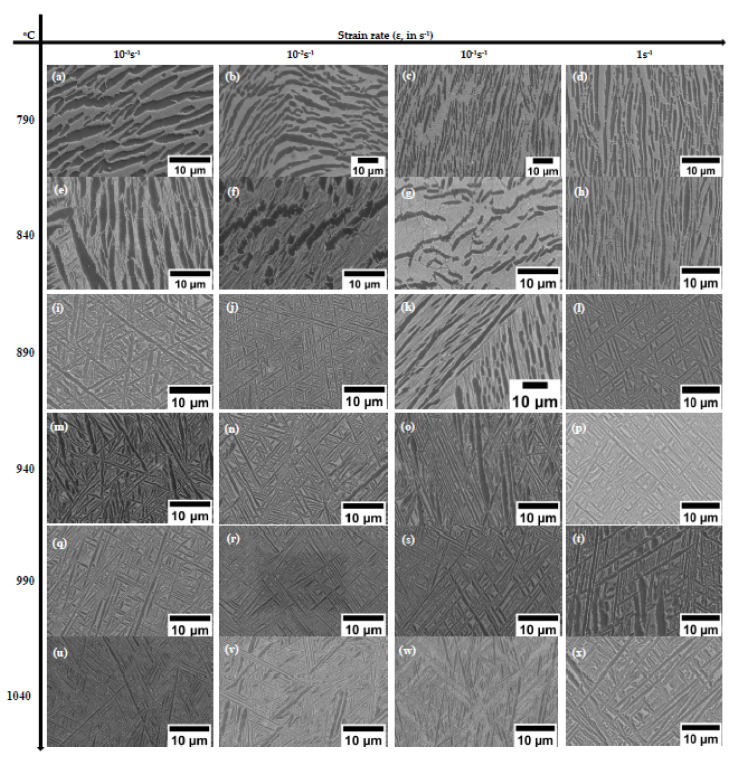
(**a**–**x**) SEM images of microstructures observed during deformations of Ti6Al4V-5Cu alloy at various temperatures and strain rates.

**Figure 10:**
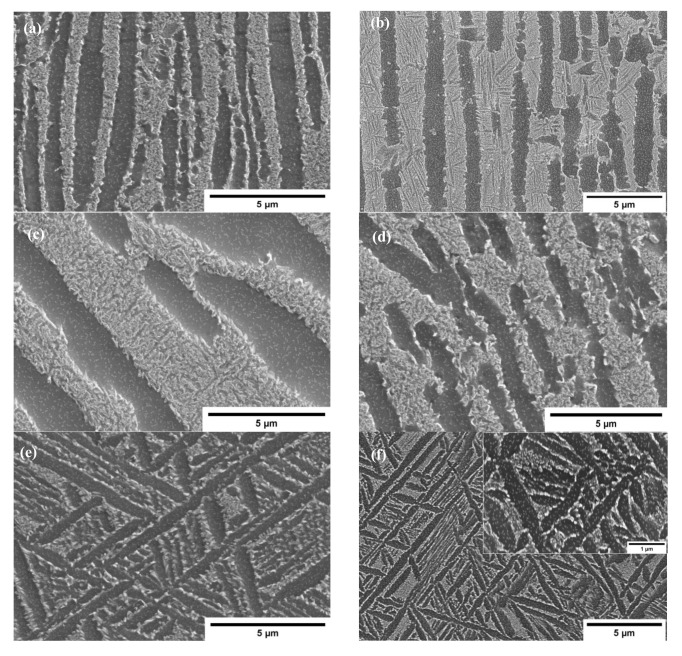
Typical SEM microstructure images of Ti6Al4V-5Cu alloy observed at high magnification during hot deformations at different temperatures and strain rates of (**a**) 840 °C and 1 s^−1^, (**b**) 840 and 10^−1^ s^−1^, (**c**) 790 °C and 10 s^−1^, (**d**) 840 °C and 10 s^−1^, (**e**) 890 °C and 1 s^−1^, and (**f**) 940 °C and 10^−2^ s^−1^.

The microstructural evolutions that occurred on Ti6Al4V-5Cu alloy deformed under different deformation conditions are shown in Figure 9 and Figure 10, which were obtained at strain of 0.4. Figure 9a shows different sized globularized α phases during deformation at 790 °C and in a strain rate of 10^−3^ s^−1^, while microstructure of the alloy compressed at 790 °C and 10^−2^ s^−1^ (Figure 9b) consisted of kinked and broken α phases with wider β phase thickness. As strain rate increased, the kinked structure became straight and elongated; in turn, its phase length increased, but width decreased. The other feature of microstructure observed after deformation at 790 °C and strain rate of 10^−1^ s^−1^ (Figure 9c) clearly indicated that the lateral dimension of α phase became smaller and the β phase began to advance and segregate into the neighboring α phase due to the increase of strain rate. Hence, the elongated α phases are broken and overwhelmed by β phases. This behavior make the α phase to conform round in shape. Such conditions indicated globularization phenomenon was undertaken. Figure 9e–h shows the microstructures of Ti6Al4V-5Cu alloy deformed at 840 °C in various strain rates. At a strain rate of 10^−3^ s^−1^, the α phases were found as elongated in shape, and the matrix of the β phase was partitioned into thin platelets and lamellar colonies inside the microstructure (Figure 9e). The thin platelets and lamellar colonies having Cu as major phase constituent created a network of β phase that suspended and floated on α phase. Under this deformation condition, the volume fraction of the β phase was calculated as 19.7% (~20%). During strain rate of 10^−2^s^−1^, an equaxed microstructure were formed (Figure 9f). Highly equaxed microstructures with densely concentrated β-phase morphology were observed during deformation at strain rate of 10^−1^s^−1^ (Figure 9g). With the increase of strain rate, a set of β phase matrix began interconnected and intermixed together so as to produce a microstructure feature, as shown in Figure 9h. It can be easily noticed that the alloy was mainly composed of β phase, which had contributed significantly to the plastic deformability of Ti6Al4V-5Cu alloy. A microscope image revealed that Ti_2_Cu precipitates, an intermetallic phase, were clearly observed during deformation at 840 °C in a strain rate of 1s^−1^. These intermetallic phases precipitated rapidly and positioned themselves at the α/β interface. Figure 9i–l shows the microstructure of alloy deformed at 890 °C in various strain rates. In the lower strain rate (Figure 9i,j), the thicker α phase of an elongated structure was positioned nearly 45° of inclination with respect to the longitudinal rolling and/or transverse direction, whereas the deformation in strain rate of 10^−1^s^−1^ revealed that Ti6Al4V-5Cu deformed specimen were composed of mixed-kind of microstructure with various α-phase morphology. Some of the α-phase was elongated in shape and directed towards transversal (TD) and/ or rolling direction (RD). Others were observed as projected remnants of α-phase, which were separated by β-phase matrix from its parent elongated α-phase structure, as seen in Figure 9k. Thus phase characteristics endorse the α-phase to be found as shorter and twisted appearance that had directionally lined-up in some sort of parallel inclinations in relation to the RD and/or TD. Such a microstructure feature would have been taken as a breakthrough for forecasting the nature of change in phase and metallurgical transformation while the as-cast structure processed through severe deformation process. At faster strain rate such as 1s^−1^ (Figure 9l), the alloy retained its microstructure observed in a slower strain rates. Figure 9m–p shows the SEM microstructure of the alloy deformed at 940 °C in various strain rates. In slower strain rate (i.e., 10^−3^s^−1^), the microstructure consisted of thicker α-phase of hcp crystalline structure with irregular rotation (Figure 9 m). The thicker elongated α-phase structures were hindered by other perpendicularly elongated α-phase structures. The thickenings of the α-phase morphology were reduced in a strain rate of 10^−2^s^−1^ (Figure 9n). However, some α (hcp) structures were observed as a remaining α- phase crystalline of the prior deformation condition. The SEM observation revealed that mixed-microstructure features of pervious deformation variables were obtained in a strain rate of 10^−1^s^−1^, as seen in Figure 9o. The microstructure had inherited a characteristic of thicker α-phase morphology with non-uniform orientation as identically obtained during 10^−3^s^−1^ strain rate, and simultaneously exhibited a microstructure feature of parallel oriented thinner α-phase structure. At higher strain rate (1s^−1^), the microstructure showed grass like morphology (Figure 9p). Figure 9q–t shows microstructure of the alloy deformed at 990 °C in various strain rates. At low strain rate such as 10^−3^s^−1^, the deformed specimen on 990°C revealed that α (hcp) phase morphology were found in straight and elongated appearance, whereas the β phase morphology had suspended on the hcp α-phase. With the increase of strain rate, the phase morphology showed variations in phase thickness. On 10^−2^s^−1^ strain rate, the α-phase thickness was found as thinner as 10^−3^s^−1^ (Figure 9q). However, during strain rate of 10^−1^s^−1^, the microstructure of deformed specimens had retained and resembled its phase thickness nearly identical to 10^−3^s^−1^. Accordingly, the thickness of α-phase was increased (Figure 9r). Straight, elongated and wider α phase structure were observed during deformation in a strain rate of 10^1^s^−1^ (Figure 9t). Figure 9u–x shows the SEM microstructure of the alloy deformed at 1040 °C in various strain rates. The microstructure of the specimen showed that some of the α phase was lamellar with longest configuration and most of the morphology comprised of thin and short phases (Figure 9u). In a strain rate of 10^−2^s^−1^, the shorter α-phase was wider in structure and found dispersedly between the longer and straight α-phases structure (Figure 9v). In a strain rate of 10^−1^s^−1^, the deformed specimen microstructure revealed that α-phase was obtained with a partially segregated β-stabilizer (Figure 9w). At faster strain rate (i.e., 10^1^s^−1^), a regularly directed α-phase crystalline structure were observed towards 45° with respect to specimen rolling direction (RD), and/or transverse direction (TD). During deformation above β transus, it is obvious that there was no apparent difference in microstructure evolution shown in lower magnification; however, the β phase morphology quite varied at higher magnification study employing scanning electron microscope (SEM), typically as seen in Figure 10.

**Figure 11:**
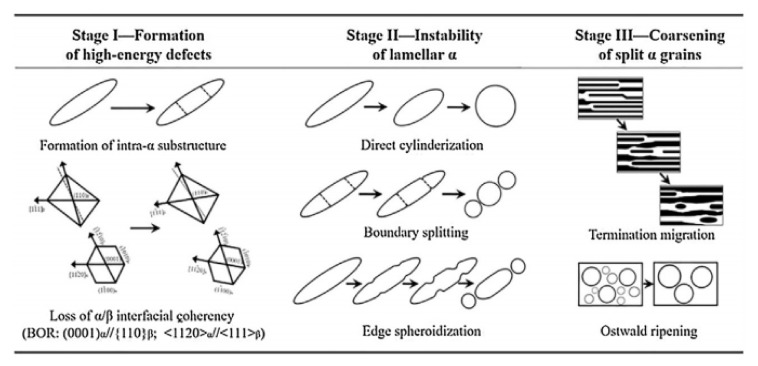
Schematic representation of globularization mechanism of α lamellar. Reprinted from Ref. [3].

The phase transformation of titanium provides the foundation for the control of microstructure. M. [3,40] described spheroidization as one of microstructure evolution mechanism by a sequence of processes involving initial loss of coherency of interphase boundaries for the development of transverse low angle boundaries within the α phase; the formation and growth of grooves at the α/β interphase grain boundary; fragmentation of lamellar platelets by the grooves; and final coarsening of the resulting small-aspect ratio α particles by diffusional processes (Figure 11).

**Figure 12:**
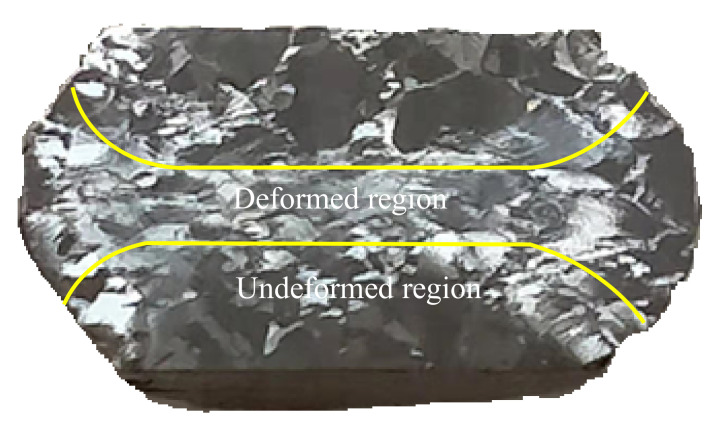
A typical Gleeble compressed sample showing highly deformed region.

The initial Ti6Al4V-5Cu alloy used for investigating the microstructural mechanism and texture study is shown in Figure 1, which is a typical lamellar transformed microstructure consisting of large prior β-grain boundaries. The microstructure is made up of thin lamellar platelets and α colonies that are projected from the interior substructure of β-grain boundaries. The electron backscattered diffraction (EBSD) analysis was conducted. One of the mechanisms that contributed to the alloy substructure transformation occurred due to the different processing conditions. The difference in deformation is possibly related to the difference in composition of titanium alloys. As shown in Figure 12, during the compression process, the central region of the sample was severely deformed. However, the region of the sample that was in close contact with the loading medium was susceptible to unstrained conditions in which grain boundaries were partially broken up. Thus, unstrained regions contributed most of the recrystallized grains during the deformation process.

**Figure 13:**
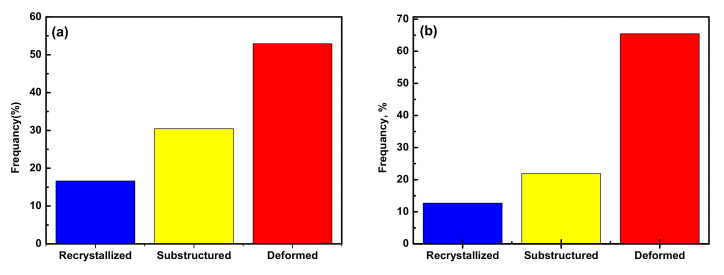
Recrystallized fraction chart for Ti6Al4V-5Cu alloy after deformation at 840 °C and different strain rates of (**a**) 0.01 s^−1^ and (**b**) 0.001 s^−1^.

**Figure 14:**
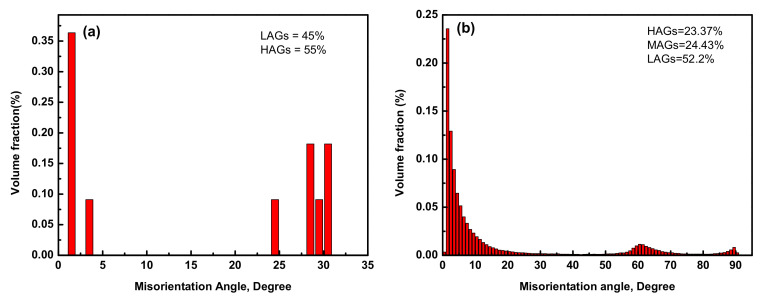
α phase misorientation angle distribution chart for deformations at different strain rates, (**a**) 0.01 s^−1^ and (**b**) 0.001 s^−1^.

**Figure 15:**
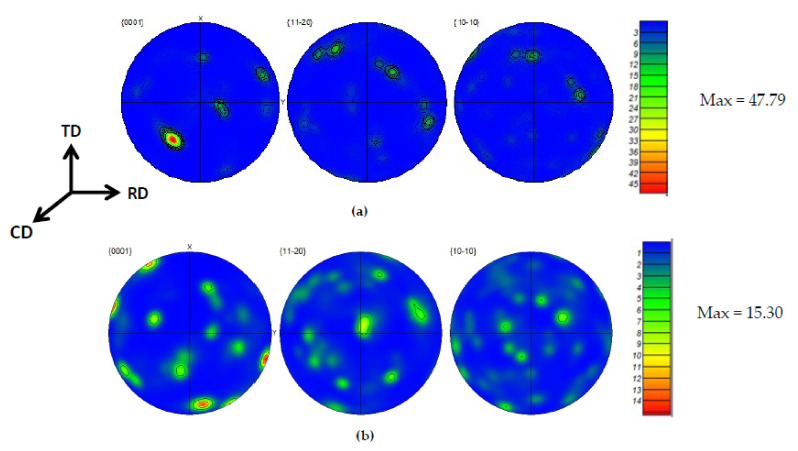
Pole figure map of α phase during deformation at 840 °C at a strain rate of (**a**) 0.01 s^−1^ and (**b**) 0.001 s^−1^.

The compression was aimed to break down the α-lamellar microstructure and develop various sets of microstructures under different deformation conditions (temperature, strain rate, and strain). The EBSD measurement results of Ti6Al4V-5Cu alloy are presented in Figure 13, Figure 14 and Figure 15. The high-angle grain boundaries (HAGs) are defined as grain boundaries with misorientation above 15°, whereas the substructures are separated by low angle grain boundaries. Thus low angle grain boundaries (LAGs) are defined as those grain boundaries with misorientation below 5°. The other grain boundaries are named as middle angle grain boundaries (MAGs) with misorientation in the range of 5° to 15°. Higher magnification using EBSD revealed that coarse elongated grains in titanium alloys contained developed substructures. Figure 13 displays the recrystallized fraction chart. The alloy was deformed with a recrystallized volume fraction of 16.63% (Figure 13a), whereas the amounts of transformed substructured and deformed grains were measured as 30.45% and 52.92%, respectively. The evolution of sub-grain and/or grain boundaries within the α phase could provide the driving force for subsequent morphology changes involving α-platelet fragmentation and spheroidization in Ti6Al4V alloy [32]. Through significant crystallographic rotation, low angle grain boundaries (LAGs) and high-angle grain boundaries (HABs) could be produced after kinking of lamellar microstructure. During deformation at a strain rate of 0.01 s^−1^, the deformed sample consisted of a 55% fraction of high-angle grain boundaries (HAGs) (Figure 14a), while the low angle grain boundaries (LAGs) constituted 45% of the overall volume fraction (Figure 14b).

As indicated in Figure 13b, as the strain rate decreased, the recrystallized volume fraction was accounted for 12.68%, whereas the transformed substructured and deformed crystallinities were 21.86% and 65.46%, respectively. Such changes in volume fraction of recrystallized, substructured, and deformed grains under different deformation conditions indicate that the deformation mechanism was triggered by dislocation movement that accelerated dynamic recrystallization (DRX) of Ti6Al4V-5Cu alloy, especially at high strain rates. At a given deformation temperature, the volume fraction of recrystallized and transformed substructured grains was increased as the deformation strain rate was increased. The evolutions of recrystallized grains were commenced at the edges of high-angle grain boundaries.

Middle angle grain boundaries (MAGs) whose misorientation ranging in 5°–15° were found during the deformation at 0.001 s^−1^ strain rate. The presence of high-angle grain boundaries (HAGs) provided information about the occurrence of high volume fractions of recrystallized grains at high strain rates. Hence, the volume fraction of HAGs for the α phase, as seen in Figure 14a, was found as 55% of grain boundaries in Ti6Al4V-5Cu alloy at a strain rate of 0.01 s^−1^. In contrast, the presence of a high volume fraction of low angle grain boundaries (Figure 14b) is attributed to the occurrences of a high volume fraction of deformed structures. At low strain rates, medium angle grain boundaries (MAGs) were one contributing factor for the formation of recrystallized grains in Ti6Al4V-5Cu alloy. The volume fraction of MAGs was measured as 24.43% at a strain rate of 0.001 s^−1^. From Figure 14b, the deformation of Ti6Al4V-5Cu alloy at a slower strain rate was accomplished by the migration of grain boundaries due to the dislocation movement. As deformation continued, a certain amount of accumulated dislocation tangles existed, and then in order to accommodate the denser dislocation phenomenon, grains rearranged themselves in the most preferential orientation through widening their inclined angles (i.e., 60°–70°) with neighboring grains to pave the way for rapid mobility and annihilation of prior accumulated dislocation tangles to further enhance alloy deformation.

Despite the type and strength of various components being dependent on the deformation mode and the processing temperature, texture has a strong effect on final mechanical properties. Texture developments on α phase lamellar are formed by the decomposition of the high-temperature β phase during the cooling of (α + β) titanium alloys. The pole figure map of the alloy is displayed in Figure 15a,b, showing the relative texture intensity obtained during deformation at strain rates of 0.01 s^−1^ and 0.001 s^−1^, respectively. The texture intensity of the alloy decreased with strain rate and vice versa. Using pole figure (PF) maps, alloy texture evolution can be elucidated. During deformation in a strain rate of 10^−2^ s^−1^, the alloy revealed that the basal {0001} fiber texture component was activated with strong texture intensity of 47.79. The PF map clearly indicates the basal texture pole was pointed at an angle of 45° tilted from compression direction (CD). To facilitate plastic deformation, at least one of the prismatic slip systems needs to be activated. On strain rate of 10^−3^ s^−1^, the deformed specimen exhibited that basal {0001} basal fiber texture components were obtained with texture intensity of 15.30. Most of the α(hcp) textured grains had positioned at 20°–80° away from RD and/or TD. Using the pole plot and pole figure (PF) map, it is clearly shown that the basal {0001} slip planes were obtained at 10° tilted from the specimen compression axis (CD). Accordingly, the activation of one of the prismatic slip systems is obvious, and for further forging deformation, it could be located perpendicular to the compression direction (CD).

The existence of such basal fiber texture pole at 80° tilted from specimen compression axis (CD) had contributed to reduce the hardening nature of texture fiber orientation during the forging process. The spread orientation of basal {0001} fiber texture towards rolling and transverse direction (RD and TD) also had a contribution to reducing deformation resistance.

## 4. Conclusions

As-cast Ti6Al4V-5Cu alloy of lamellar starting microstructure was investigated using isothermal compression testing. The features of microstructure evolution were found to be temperature and strain-rate-dependent. Based on the generated processing map (PM), true stress-strain relationship, and microstructure evolution, the following conclusions have been drawn. An initial lamellar starting microstructure with colonies initially not aligned with the metal flow direction (perpendicular to compression axis) with various degrees of strain rate sensitivity provided various morphological changes during the isothermal deformation process. In the single β phase region, power dissipation efficiency (η) reached 89% during deformation at higher temperatures and lower strain rates. Under various deformation conditions, alloy exhibited different microstructure features of β phase morphology. The deformation of alloy limited the instability region that existed in the temperature range of 800 to 930 °C at higher strain rates (>1 s^−1^). The degree of instability in the dual (α + β) phase decreased with the increase in deformation temperature and decrease in strain rate. Activation energies (Q) in the dual-phase (α + β) and single β phase regions were calculated as 175.43 and 159.03 kJ mol^−1^, respectively. The intensity of oscillatory change in alloy flow stress profiles increased as strain rate was increased, which provides an insight that work-hardening and flow-softening mechanisms were coexisted in Ti6Al4V-5Cu alloy under severe plastic deformation conditions.

## Figures and Tables

**Table 1:** Peak stress (in MPa) and corresponding true strain (in mm/mm) of Ti6Al4V-5Cu alloy hot compressed at various deformation temperatures and strain rates.

Peak Stress—True Strain Value			
Temp. (°C)	Strain Rate (s^−1^)	Peak Stress (MPa)	True Strain
790	10^−3^	67.916	0.019
	10^−2^	100.933	0.025
	10^−1^	207.742	0.025
	1	294.672	0.035
	10	420.788	0.039
840	10^−3^	38.170	0.032
	10^−2^	62.408	0.010
	10^−1^	127.975	0.025
	1	208.564	0.028
	10	323.906	0.032
890	10^−3^	23.112	0.020
	10^−2^	50.811	0.010
	10^−1^	79.159	0.010
	1	152.829	0.016
	10	241.290	0.017
940	10^−3^	15.356	0.039
	10^−2^	40.602	0.010
	10^−1^	76.360	0.013
	1	117.300	0.012
	10	271.368	0.006
990	10^−3^	11.032	0.033
	10^−2^	54.334	0.007
	10^−1^	69.108	0.008
	1	116.594	0.011
	10	223.833	0.003
1040	10^−3^	16.850	0.038
	10^−2^	36.739	0.006
	10^−1^	61.526	0.012
	1	92.993	0.011
	10	181.240	0.002
